# Impact of Microgroove Shape on Flat Miniature Heat Pipe Efficiency

**DOI:** 10.3390/e20010044

**Published:** 2018-01-11

**Authors:** François Ternet, Hasna Louahlia-Gualous, Stéphane Le Masson

**Affiliations:** 1 Normandy University, Unicaen-LUSAC, 120 Rue de L’exode, 50000 Saint Lô, France; 2 Orange Labs, 2 Avenue Pierre Marzin, 22307 Lannion, France

**Keywords:** heat pipe, electronic cooling, energy efficiency, heat transport limitation, sizing procedure

## Abstract

Miniature heat pipes are considered to be an innovative solution able to dissipate high heat with low working fluid fill charge, provide automatic temperature control, and operate with minimum energy consumption and low noise levels. A theoretical analysis on heat pipe thermal performance using Deionized water or *n*-pentane as the working fluid has been carried out. Analysis on the maximum heat and capillary limitation is conducted for three microgroove cross sections: rectangular, triangular, and trapezoidal. The effect of microgroove height and width, effective length, trapezoidal microgroove inclination angle, and microgroove shape on heat pipe performance is analysed. Theoretical and experimental investigations of the heat pipes’ heat transport limitations and thermal resistances are conducted.

## 1. Introduction

Using micro-heat pipes in electronic cooling offers appreciable advantages, such as a high heat transfer coefficient, low working fluid quantities, and high compactness [1]. Therefore, various prototypes of micro-cooling systems and numerous studies on microscale two-phase flows are of interest to scientists and engineers [2,3,4,5]. From the open literature review, it appears that a high-efficiency cooling system with low electrical consumption and low environmental impact needs to be investigated. Miniature heat pipes are considered to be an innovative passive cooling solution able to dissipate high heat fluxes with low working fluid charge. They automatically control the operating temperature, and operate with a minimum energy consumption and less noise [5,6,7,8].

**Figure 1:**
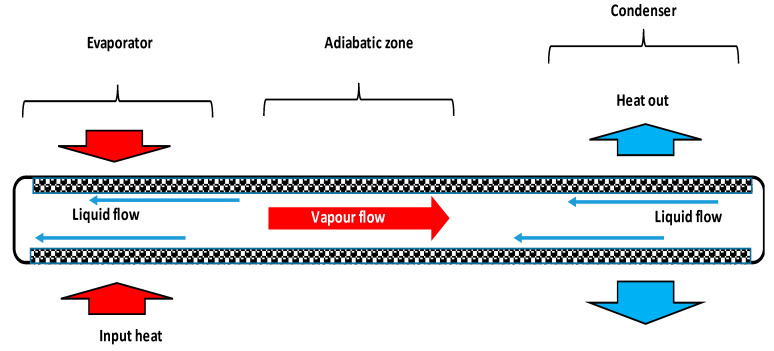
Heat transfer in a heat pipe.

Due to their ability to improve component and system energy efficiency, micro-heat pipes are considered to be the appropriate cooling option for reducing refrigerant charge and greenhouse gas emissions. Indeed, the small channels’ size in heat exchangers constitutes an innovative method providing effectiveness, compactness, low thermal resistance, and environmental protection by the reduction of refrigerant charge. Micro-heat pipes are used in many applications because they work without electricity. Their performance is related to the capillary limit, vapor flow limit, liquid–vapor interface, and to heat and mass transfer in the liquid and vapor flows. In the heat pipe (Figure 1), the working fluid is vaporized in the evaporator where the input heat load is applied. Vapor moves through the adiabatic section to the condenser where it is liquefied. After condensation, liquid is driven from the condenser to the evaporator under the pressure of capillary force generated in the microgrooves.

Various research studies in the available literature are focused on heat pipes with microgrooves, but there have been few investigations into the effect of microgroove shape on the maximum heat flux and capillary pressure. Cotter [9] proposed the concept of a wickless heat pipe, which was designed to obtain uniform temperature distribution for electronic chips. Peterson [9] used the Laplace–Young equation to describe the fluid dynamics in heat pipes and found that longitudinal microgroove design was a critical parameter to keep the uniform temperature distribution and to maintain the operating temperature. Jiao et al. [10] developed a mathematical model to predict the contact angle effect, thin film profile, and heat flux distribution in heat pipes with trapezoidal microgrooves. They found that in the evaporator section, heat transfer trough the thin film region decreases with superheat. Faghri et al. [11] conducted an experimental and theoretical study on the maximum heat transfer for flat miniature heat pipes with diagonal trapezoidal and rectangular microcapillary grooves. They found that the maximum heat flux of the heat pipe with rectangular grooves exceeds 90 W/cm^2^ in the horizontal orientation and 150 W/cm^2^ in the vertical orientation. Khrustalev and Faghri [12] developed a one-dimensional mathematical model for heat transfer during liquid evaporation in a porous structure at high heat flux. The model characterizes evaporation heat transfer and the location of the liquid–vapor interface. Suh [13] analyzed liquid and vapor flows in trapezoidal and sinusoidal microgrooves taking into account the shear stress effect along the liquid–vapor interface. A modified correlation for liquid friction is proposed for trapezoidal and sinusoidal microgrooves.

Based on the previous works, the present study is focused on the prediction of heat pipe thermal efficiency under various operating conditions. An analytical model for the heat pipe with various microgroove sizes and shapes is developed to predict the maximum heat flux, pressure losses, and capillary radius. A number of factors influencing heat transfer in the heat pipes are studied, compared, and discussed. Various microgroove shapes and working fluids are tested in order to highlight their effects on heat pipe efficiency.

## 2. Heat Pipe Analytical Model

The model considers a microgrooved heat pipe where the liquid flows along the heat exchange surface and the vapor flows in the core space. Various microgroove shapes (rectangular, trapezoidal, and triangular) are considered. The studied heat pipe consists of three thermal zones: condensate zone, adiabatic zone, and evaporative zone. In each zone, pressure, momentum, shear stress, and capillary are considered as the driving forces for the liquid flow and vapor flow. These forces are dependent on the surface tension, meniscus curvature radius, heat pipe surface, and the flow volume. The curvature radius of the liquid film is related to the capillary pressure, which is variable along the axial direction from the condenser end to the evaporator end. It is a function of the liquid and vapor pressures in the heat pipe evaporator, condenser, and adiabatic zones. The maximum capillary pressure rises due to the surface tension effect in the grooves; it is defined by the Laplace–Young equation as

(1) $$∆P_{cap,max}=\frac{\sigma }{R_{m}}$$

where σ is the surface tension; and $R_{m}$ is the meniscus radius, which depends on the heat pipe microgroove shapes.

**Figure 2:**
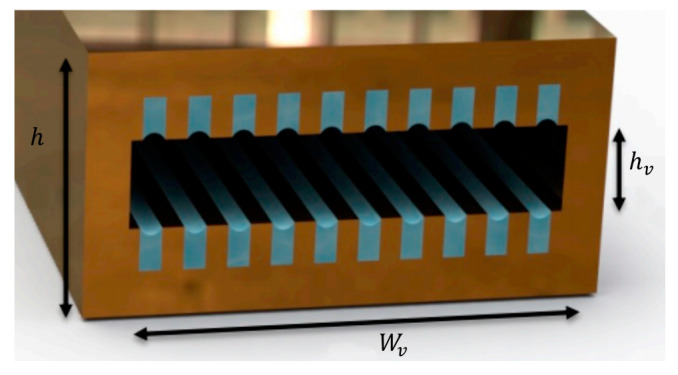
Cross section of the flat heat pipe.

**Figure 3:**
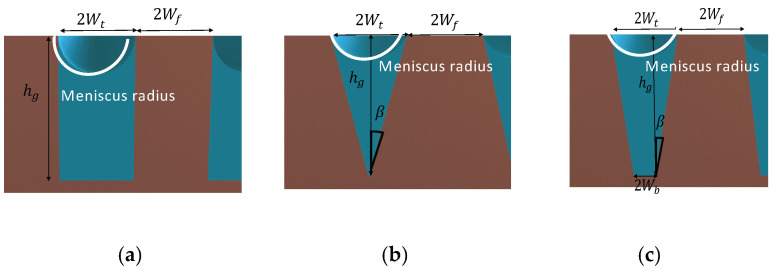
Microgroove shape in the heat pipe: (**a**) rectangular, (**b**) triangular, (**c**) trapezoidal.

Figure 2 shows an axially microgrooved flat miniature heat pipe used in this work. The heat pipe width and height are designated W and h, respectively. The vapor cross section width and height are designated *W_v_* and *h_v_*, respectively. The geometrical parameters for triangular, rectangular, and trapezoidal microgrooves are shown in Figure 3. The microgrooves’ top width and depth are designated 2*W_t_* and *h_g_*, respectively. For trapezoidal microgrooves, 2*W_b_* is the bottom width.

### 2.1. Meniscus Radius Expressions

**Figure 4:**
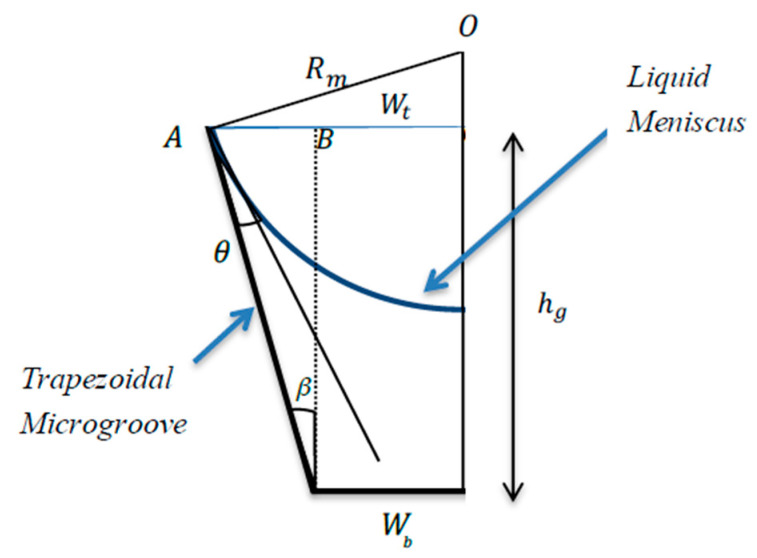
Trapezoidal microgroove.

Figure 4 shows a schematic geometry of a trapezoidal microgroove half cross section. Here, $h_{g}$ denotes the microgroove depth, β is the inclination angle, and θ is the meniscus contact angle. The top width of the microgroove is defined as

(2) $$\left|AO^{\prime }\right|=R_{m}\cos\left(\beta +\theta \right)=h_{g}\tan\left(\beta \right)+W_{b}.\;$$

The meniscus radius expression deduced from Equation (2) is

(3) $$R_{m}=\frac{h_{g}\tan\left(\beta \right)+W_{b}}{\cos\left(\beta +\theta \right)}\;.$$

For a rectangular microgroove, where β=0, the meniscus radius is expressed as

(4) $$R_{m}=\frac{W_{b}}{\cos\left(\theta \right)}\;.$$

For a triangular microgroove, $W_{b}=0$, and Equation (3) becomes

(5) $$R_{m}=\frac{h_{g}\tan\left(\beta \right)}{\cos\left(\beta +\theta \right)}\;.$$

### 2.2. Capillary Limit

For a flat grooved heat pipe, the capillary pressure needs to be higher than the sum of all the pressure losses in the liquid and vapor flows.

(6) $$∆P_{cap,max}\geq \;∆P_{l}+∆P_{v}\;$$

The liquid and vapor pressure losses increase with input heat flux. The capillary limit is reached when the sum of all the pressure losses become equal to the maximum capillary pressure.

#### 2.2.1. Liquid Flow Pressure Loss 

As defined by Khrustalev and Faghri [13], the pressure loss in the liquid flow is expressed by taking into account the groove inclination (ϕ≠0) and the axial grooves (ϕ=0) inside the heat pipe. For an inclined heat pipe, the modified momentum conservation equation for liquid flow is

(7) $$\frac{dP_{l}}{dz}=\mu_{l}\frac{2\left(f_{l}Re_{l}\right)}{\cos\left(\phi \right)D_{hl}^{2}}U_{l}-\rho_{l}\;g\;\sin\left(\alpha \right)$$

where $U_{l}$ is the average liquid velocity, $\rho_{l}$ is the liquid density, ϕ is the groove inclination angle, $D_{hl}$ is the hydraulic diameter of the liquid cross sections, and α is the heat pipe inclination angle. The liquid Reynolds number is determined by

(8) $$Re_{l}=\frac{\rho_{l}U_{l}D_{hl}}{\mu_{l}}.$$

The liquid friction factor–Reynolds number product ($f_{l}Re_{l}$) is correlated with respect to the groove shape. For the rectangular microgroove, the friction-Reynolds number coefficient is defined as

(9) $${\left(fRe\right)}_{l}={\left(f\;Re\right)}_{l0}\left(1+\frac{4N}{3\;\pi }\;{\left(\frac{W_{t}}{D_{hl}}\right)}^{3}\;\frac{\nu_{v}}{\nu_{l}}{\left(fRe\right)}_{v}\right)\left(1-1.971\;\exp\left(-\frac{\pi \;h_{g}}{2W_{t}}\right)\right)$$

where ${\left(f\;Re\right)}_{l0}$, for liquid flow only with no liquid–vapor interaction, is defined [7] as

(10) $${\left(f\;Re\right)}_{l0}=\frac{8\;h_{g}^{2}}{W_{t}^{2}{\left(1+\frac{h_{g}}{W_{t}}\right)}^{2}\left(\frac{1}{3}-\frac{64W_{t}}{\pi^{5}\;h_{g}}\tan h(\frac{\pi \;h_{g}}{2W_{t}}\right)}.$$

The liquid hydraulic diameter is estimated using

(11) $$D_{hl}=4\;\frac{2W_{t}h_{g}-\frac{\pi R_{m}^{2}}{2}+[R_{m}^{2}\theta +W_{t}R_{m}\sin\left(\theta \right)]}{\left(2\;W_{t}+2\;h_{g}\right)}.$$

For the trapezoidal microgrooves, Suh et al. [14] have proposed the use of the following expression:

(12) $${\left(fRe\right)}_{l}=B{\left(fRe\right)}_{l0}\;\left[1-\left(1-1.971\;\exp\left(\frac{\pi \;h_{e}}{2W_{c}}\right)\right)\;E\;{\left(f\;Re\right)}_{v}\;\frac{\;h_{e}}{6D_{hv}}\;\frac{\mu_{v}\;U_{v}}{\mu_{l}\;U_{l}}\;{\left(\frac{W_{c}}{\;h_{e}}\right)}^{2}\right]$$

where

(13) $$B=1.44-\;\frac{0.84}{1+\sin\left(\theta \right)}\left(1-0.19\;\sqrt{1-{\left(\frac{W_{b}}{W_{t}}\right)}^{2}}\right),$$

(14) $$E=-1.2+1.1\left(\frac{W_{t}}{\;h_{g}}-\theta \right)+1.6\exp{\left(\frac{W_{b}}{W_{t}}\right)}^{3}-0.45\sqrt{\frac{W_{t}}{W_{t}+W_{f}}}\;\;\;+\frac{h_{g}}{h_{v}}\left(1.6-0.77\frac{W_{t}}{\;h_{g}}-1.6\exp\left(\;{\left(\frac{W_{b}}{W_{t}}\right)}^{3}\right)+1.3\theta \right).$$

The liquid depth and width are determined from

(15) $$h_{e}=h_{g}-R_{m}(1-\sin\left(\theta +\beta \right)),\;$$

(16) $$W_{c}=W_{t}+\frac{1}{2}h_{g}\tan\left(\beta \right).\;$$

The liquid hydraulic diameter for the trapezoidal groove is defined as

(17) $$D_{hl}=\;\frac{\left(2W_{t}-h_{g}\;\tan\left(\beta \right))\;h_{g}-\pi R_{m}^{2}+2\;[R_{m}^{2}\left(\theta +\alpha \right)+W_{t}R_{m}\sin\left(\theta +\alpha \right)\right]}{\frac{h_{g}}{\cos\left(\beta \right)}+W_{b}}.$$

**Figure 5:**
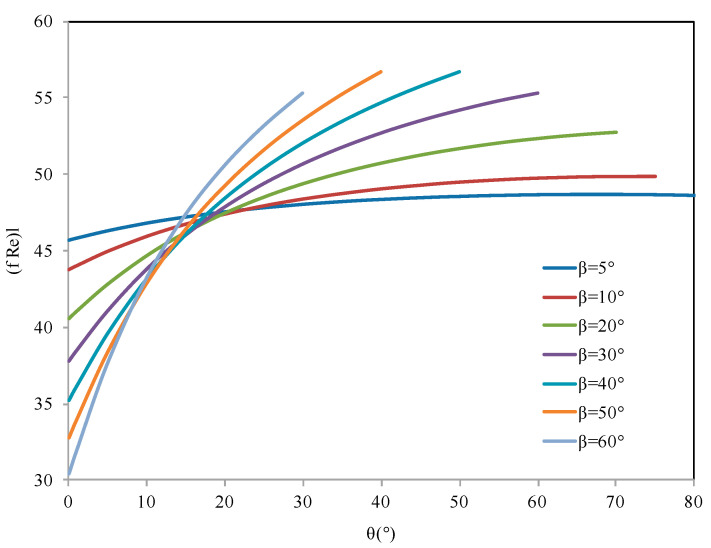
Friction-Reynolds number coefficient for liquid flow in triangular grooves.

For triangular microgrooves, the friction-Reynolds number factor $f_{l}Re_{l}$ is deduced from the analytical results of Ayyaswamy et al. [15], defined for channel half angles from 5 to 60° and contact angles varying from 0.1 to 85°. Ayyaswamy et al. [15] have presented $f_{l}Re_{l}\;$ in the form of tables. Based on the values obtained by these authors, Figure 5 shows the variation of $f_{l}Re_{l}$ for different values of groove half angle and contact angle. For each fixed groove half angle, $f_{l}Re_{l}$ increases with increase of the contact angle. The impact of the contact angle on the $f_{l}Re_{l}$ value is high for high values of β, and becomes very low for β lower than 10°. For each fixed pair of values for β and θ, $f_{l}Re_{l}$ can be estimated using a polynomial interpolation of the results presented in Figure 5.

The liquid hydraulic diameter is described as follows:

(18) $$D_{hl}=\frac{-h_{g}^{2}\;\tan\left(\beta \right)-\pi R_{m}^{2}+2\;R_{m}^{2}\left(\theta +\beta \right)}{h_{g}/\cos\left(\beta \right)}.$$

#### 2.2.2. Vapor Flow Pressure Loss 

The vapor pressure loss is defined as the sum of the viscous, volume, and inertial pressure losses along the flow direction. Variation of the vapor pressure in the radial and angular directions is negligible. The vapor pressure is defined as

(19) $$\frac{d\;P_{v}}{dz}=-\mu_{v}\frac{2\left(f_{v}Re_{v}\right)}{D_{hv}^{2}}U_{v}-\rho_{v}g\sin(\alpha )-\frac{d\;}{dz}\left(\rho_{v}\;\beta \;U_{v}^{2}\right)$$

where $U_{v}$ is the average vapor flow velocity, $D_{hv}$ is the vapor zone hydraulic diameter, and β represents the vapor moment coefficient equal to 4/3 for laminar vapor flow as defined by Faghri et al. [16]. The vapor Reynolds number is defined as

(20) $$Re_{v}=\frac{\rho_{v}U_{v}D_{hv}}{\mu_{v}}\;,$$

(21) $$\;D_{hv}=\frac{\left(W_{f}+W_{t}\right)Nh_{v}}{N\;W_{f}+2h_{v}}.$$

For rectangular and triangular grooves, the friction-Reynolds number coefficient is defined as follows:

(22) $$f_{v}Re_{v}=24\;\left(1-1.3553C+1.9467C^{2}-1.7012C^{3}+0.9564C^{4}-0.2537C^{5}\right)$$

with $C=\min\left(\frac{h_{v}}{W_{v}},\frac{W_{v}}{h_{v}}\right)$, where *W_v_* and *h_v_* are the vapor width and depth defined as

(23) $$W_{v}=N\left(W_{t}+W_{f}\right),$$

(24) $$h_{v}=h_{t}-2h_{w}-2h_{g}.$$

For trapezoidal microgrooves, Suh et al. [14] investigated liquid and vapor flows in trapezoidal grooves and proposed the following correlation for the friction–Reynolds number coefficient:

(25) $${\left(fRe\right)}_{v}=\left(-0.94+3.8\;e^{\frac{\pi }{2}}+\frac{11.8}{1+\sin\theta }\right)+{\left(\frac{W_{t}}{W_{t}+W_{f}}\right)}^{2}\left(52+4.6e^{\frac{\pi }{2}}+\frac{0.89}{1+\sin\theta }\right).$$

### 2.3. Heat Pipe Effective Length and Maximum Heat

The effective length is used to characterize the heat pipe zone of fluid circulation with a constant heat flux. It is defined by the following expression [16]:

(26) $$L_{eff}=\int_{0}^{L_{t}}\int_{0}^{W_{v}}f_{n}\left(x,z\right)dxdz\;$$

where $L_{t}$ is the total heat pipe length, and $W_{v}$ is the heat pipe vapor width. The function $f_{n}\left(x,z\right)$ is piecewise defined for each zone of the heat pipe, assuming that local heat flux is dissipated following a linear evolution. 

For the evaporation zone $\left(0\leq z\leq L_{e}\right)$:

(27) $$f_{n}\left(z\right)=\frac{z}{W_{v}L_{e}};$$

For the adiabatic zone $\left(L_{e}\leq z\leq L_{e}+L_{a}\right)$:

(28) $$f_{n}\left(z\right)=\frac{1}{W_{v}};$$

For the condensation zone $\left(L_{e}+L_{a}\leq z\leq L_{t}\right)$:

(29) $$f_{n}\left(z\right)=\frac{L_{t}-z}{W_{v}L_{c}}\;$$

where $L_{c}$ is the condenser heat pipe length, and $L_{a}$ is the adiabatic length of the heat pipe.

The effective length is given as the following:

(30) $$L_{eff}=\frac{L_{e}}{2}+L_{a}+\frac{L_{c}}{2}\;.$$

Heat flux in the heat pipe condenser and evaporator is nonuniform and is related to the phase change rate and distribution of the liquid film thickness. It is constant in the adiabatic zone. The maximum heat flux that could be dissipated by the heat pipe is

(31) $$\phi_{max}=\frac{1}{L_{eff}}\;\overset{L_{t}}{\underset{0}{\int }}\phi \left(z\right)dz\;$$

where ϕ(z) represents the local axial heat flux calculated by taking into account the liquid and vapor conservation momentum equations, energy, and mass balance equations. It is defined as

(32) $$\phi \left(z\right)\;=U_{l}\;\rho_{l}\;N\;A_{l}\;h_{lv}$$

where $U_{l}$ and $A_{l}$ are the velocity and cross section of the liquid flow along the heat pipe, respectively, and $h_{lv}$ represents fluid latent heat. Considering the Laplace–Young expression, the liquid velocity is related to the vapor and liquid pressure drops by

(33) $$-\frac{d}{dz}\left(\frac{\sigma }{R_{m}}\right)=\frac{d\left(P_{l}-P_{v}\right)}{dz}.$$

Introducing the liquid and vapor conservation momentum equations, the Laplace–Young expression could be written as

(34) $$-\frac{d}{dz}\left(\frac{\sigma }{R_{m}}\right)=(\rho_{v}-\rho_{l})g\sin\left(\alpha \right)+2\left(\mu_{l}\frac{\left(f_{l}Re_{l}\right)}{\cos\left(\phi \right)D_{hl}^{2}}U_{l}+\mu_{v}\frac{\left(f_{v}Re_{v}\right)}{D_{hv}^{2}}U_{v}\right)+\frac{d\;}{dz}\left(\rho_{v}\;\beta \;U_{v}^{2}\right)$$

where

(35) $$U_{l}=\frac{\phi \left(z\right)}{\rho_{l}\;A_{l}\;h_{lv}}\;.$$

Considering mass conservation equation in both phases,

(36) $${\dot{m}}_{t}=U_{v}\rho_{v}A_{v}\;=\;N\;\rho_{l}\;A_{l}U_{l}.\;$$

Vapor velocity could be written as

(37) $$U_{v}=\frac{\;N\;\rho_{l}\;A_{l}}{\rho_{v}A_{v}}U_{l}.\;$$

Using Equations (35) and (37), Equation (35) could be written as

(38) $$\begin{array}{cc} -\frac{d}{dz}\left(\frac{\sigma }{R_{m}}\right)= & (\rho_{v}-\rho_{l})gz\sin\left(\alpha \right)+\frac{\beta }{\rho_{v}}\frac{d\;}{dz}{\left(\frac{N}{A_{v}}\frac{\phi \left(z\right)}{\;h_{lv}}\right)}^{2}\\ & \;+2\frac{\phi \left(z\right)}{\rho_{l}\;N\;A_{l}\;h_{lv}}\left(\mu_{l}\frac{\left(f_{l}Re_{l}\right)}{\cos\left(\phi \right)D_{hl}^{2}}+\mu_{v}\frac{\left(f_{v}Re_{v}\right)}{D_{hv}^{2}}\frac{\;N\;\rho_{l}\;A_{l}}{\rho_{v}A_{v}}\right). \end{array}$$

We integrate this expression along the total length, assuming that the meniscus radius at the end of the condensation zone $\left(z=L_{t}\right)\;$: $R_{m}\left(z=L_{t}\right)=\infty$ because the microgrooves are filled. At the evaporator end cap (z=0), the meniscus radius is assumed to be at a minimum $R_{m,min}$. The previous equation becomes

(39) $$\begin{array}{cc} \frac{\sigma }{R_{m}\left(z=L_{t}\right)}-\frac{\sigma }{R_{m}\left(z=0\right)} & =(\rho_{v}-\rho_{l})g\;L_{t}\sin(\alpha )\\ & +\frac{2}{\rho_{l}\;A_{l}\;h_{lv}}\left(\frac{\mu_{l}\left(f_{l}Re_{l}\right)}{\cos\left(\phi \right)D_{hl}^{2}}+\frac{\mu_{v}\left(f_{v}Re_{v}\right)}{D_{hv}^{2}}\frac{\;N\;\rho_{l}\;A_{l}}{\rho_{v}A_{v}}\right)\phi_{max}L_{eff}. \end{array}$$

The maximum heat flux can be written as

(40) $$\phi_{max}=\frac{(\rho_{l}-\rho_{v})g\;L_{t}\sin\left(\alpha \right)-\frac{\sigma }{R_{m,min}}}{\frac{2}{\rho_{l}\;N\;A_{l}\;h_{lv}}\left(\mu_{l}\frac{\left(f_{l}Re_{l}\right)}{\cos\left(\phi \right)D_{hl}^{2}}+\mu_{v}\frac{\left(f_{v}Re_{v}\right)}{D_{hv}^{2}}\frac{\;N\;\rho_{l}\;A_{l}}{\rho_{v}A_{v}}\right)L_{eff}}.$$

### 2.4. Solution Procedures

The governing equations, combined with the boundary conditions, are solved through an iterative procedure. To start the calculation, the input heat load, shape and number of microgrooves, heat pipe size, and working fluid nature are specified. A modeling Fortran program was created following this process:

**Step 1**: The input parameters are imposed, and the working fluid thermophysical properties are calculated. The saturation temperature is deduced from the pressure and the heat pipe size and orientation;
**Step 2**: An initial value of the heat flux is given;
**Step 3**: Selection of the microgroove number;
**Step 4**: Calculation of the maximum heat flux;
**Step 5**: Calculation of the heat transport limits;
**Step 6**: Calculation of the hydraulic limits;
**Step 7**: Calculation of the fluid velocities;
**Step 8**: Calculation of the pressure differences;
**Step 9**: Calculation of the boiling limitation;
**Step 10**: Check the convergence criterion calculations. If this criterion is not satisfied, a new value will be estimated by the secant method, taking into account the difference obtained on the estimation of the new value of the heat flux and the value imposed at Step 2;
**Step 11**: The calculation loop is repeated until the error is less than the imposed criterion.

Calculations were stopped when total condensation was reached in the tube or the vapor quality became lower than 1%.

## 3. Results

There are many parameters affecting the heat transfer and thermal efficiency of heat pipes. In this study, analysis is focused on the determination of the geometrical parameters and their effect on the maximum heat flux, used as the first index of the thermal performance of the heat pipe. The second index concerns the capillary pressure, which must be higher than the sum of the pressure drops in the liquid and vapor flows. The calculation was firstly started by using *n*-pentane as the working fluid and 55 °C as the heat pipe working temperature. The flat heat pipe length, width, and height are 63 mm, 10 mm, and 6 mm, respectively. The evaporator length is 13 mm. Optimization of the geometrical parameters of the flat heat pipe for dissipation of an input heat load of 15 W was conducted for different microgroove configurations.

### 3.1. Determination of the Heat Pipe Design

#### 3.1.1. Flat Heat Pipe with Rectangular Microgrooves

**Figure 6:**
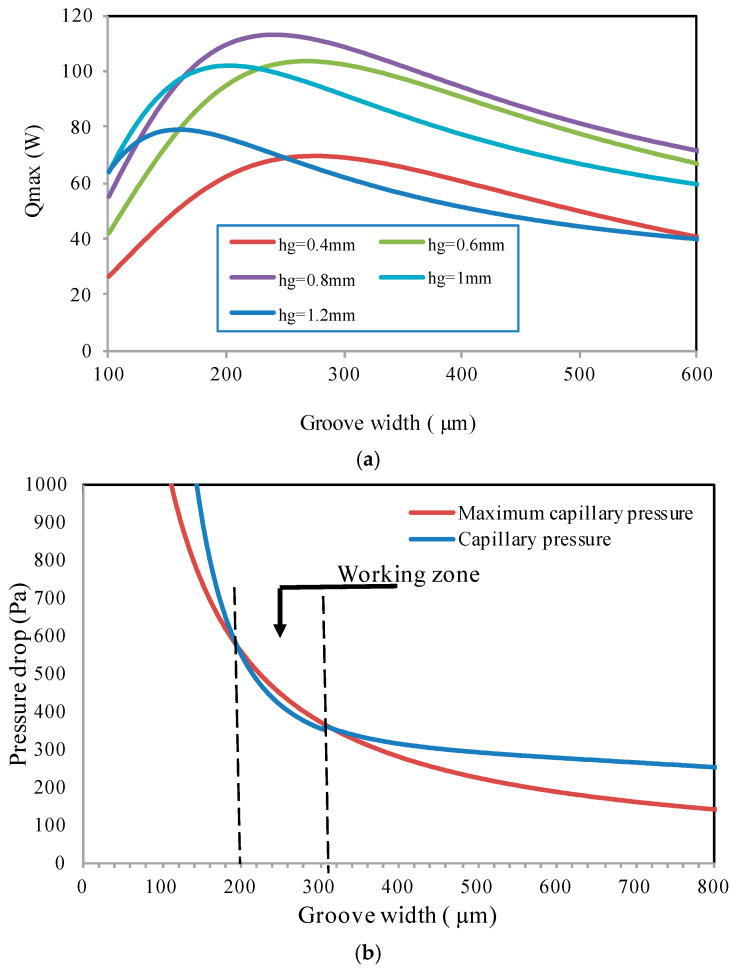
Flat heat pipe with rectangular grooves: (**a**) maximum heat flux, (**b**) pressure differences.

In this section, the effect of groove width and depth for a flat heat pipe is studied. The number of grooves is fixed to 20 per section. The maximum heat flux and total pressure are calculated for different fixed values of groove depth (400, 600, 800, 1000, and 1200 μm). The groove width is varied in the range of 100–600 μm. Figure 6a compares the maximum heat flux calculated for the imposed heat load of 15 W. *N*-pentane at 55 °C was used as the working fluid in the heat pipe. It can be seen that the maximum heat flux increases with the microgroove width for low values of *W_t_*, where the capillary pressure decreases (Figure 6a). This figure presents the heat pipe working zone where the maximum capillary pressure is greater than the sum of the liquid and vapor pressure drops. It shows that the maximum heat flux decreases gradually by increasing the groove width, where the total pressure decreases slightly and becomes greater than the maximum capillary pressure, as shown by Figure 6b.

**Figure 7:**
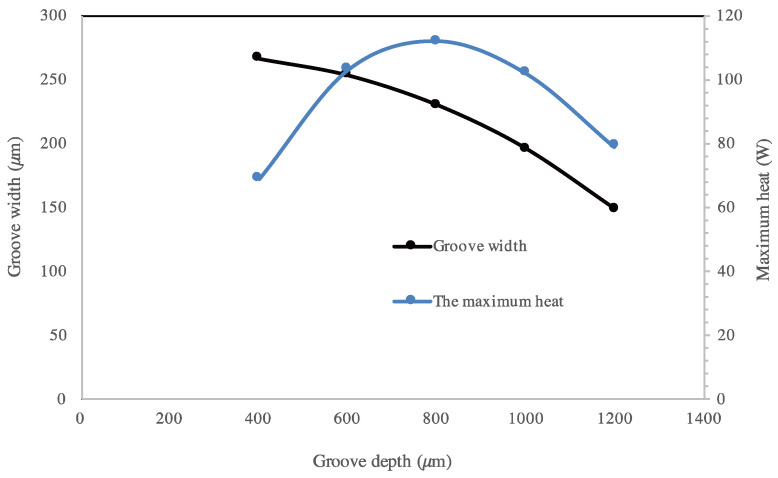
Maximum heat flux for a flat heat pipe with rectangular microgrooves.

Figure 6b shows that, for the flat heat pipe with rectangular microgrooves, the working zone is obtained for a microgroove width ranging from 200 to 300 μm where the maximum capillary pressure is higher than the sum of the liquid and vapor pressures. Results presented in Figure 6b are obtained for a microgroove depth of 800 μm. In the working zone, the total pressure is sufficient to drive liquid from the condenser to the evaporator, wetting the heat exchange surface. Respecting the working heat pipe capillary condition, Figure 7 shows the maximum heat flux for different groove sizes. The highest value of the maximum heat flux is obtained for a groove depth ranging from 700 to 900 μm and groove width ranging from 270 to 290 μm. These parameters are used as the optimum ranges for choosing the size of rectangular grooves.

#### 3.1.2. Flat Heat Pipe with Trapezoidal Microgrooves

**Figure 8:**
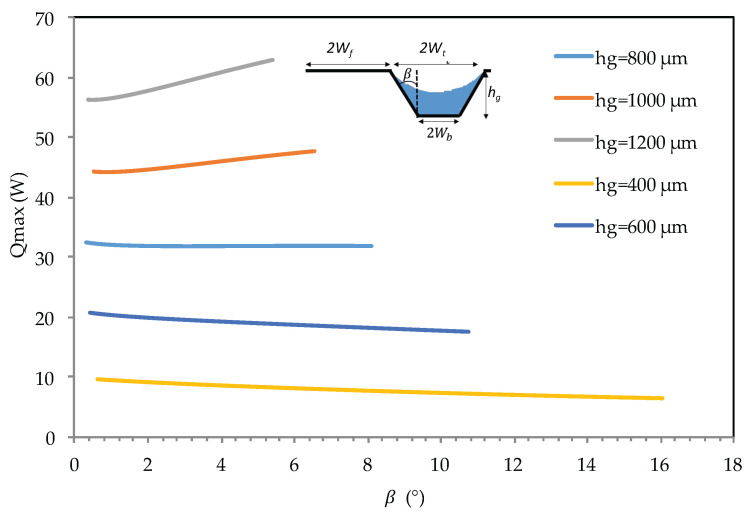
Impact of β on the maximum heat for a flat heat pipe with trapezoidal grooves.

For an input heat load of 15 W, Figure 8 compares the maximum heat for flat heat pipes with trapezoidal microgrooves. Calculations are conducted for different values of angle β. The top width of the microgrooves, $2W_{t}$, is 280 μm. The number of grooves is fixed at 20 for all calculations. The heat pipe is in a horizontal orientation (α=0°). Figure 8 shows that the maximum heat is influenced by the angle β for the deeper grooves (*h_g_* > 800 μm) where the variation of the liquid section is more influenced by the variation of β. For the same β, it can be seen that the maximum heat is increased from 10 to 60 W for heat pipe microgroove depth varying from 400 to 1200 μm.

#### 3.1.3. Influence of the Microgroove Shape on the Maximum Heat Flux

One of the important mechanisms for improving the condensation and evaporation heat transfer is the corners in the microchannels that retain the major part of the liquid flow. Microgroove shape influences the two-phase flow distribution in the heat pipe under surface tension forces. The influence of the microgroove shape on the heat pipe efficiency is highlighted by studying different microgrooves with the same cross-sectional area or the same hydraulic diameter. For the same microgroove cross-sectional area, calculations are conducted for three configurations: (i) rectangular microgroove of 270 × 800 µm^2^; (ii) isosceles triangular microgroove where the remaining side is 280 µm and the triangle height is 1600 µm; and (iii) trapezoidal microgrooves with a height of 1600 µm, top width of 280 µm, and angle *β* of 4°.

**Figure 9:**
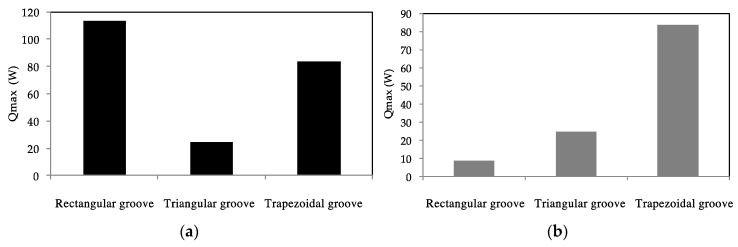
Comparison of heat pipe efficiency for: (**a**) the same cross-sectional area; (**b**) the same hydraulic diameter.

Under the same conditions, Figure 9a compares the maximum heat for each configuration with the same cross-sectional area. According to this figure, it can be seen that the lowest heat transfer is for the microgrooves with a triangular cross section. On the other hand, the highest maximum heat is obtained for the microgrooves with a rectangular cross section. It is interesting to note that, for the same hydraulic diameter, the highest maximum heat is obtained for the trapezoidal cross section (Figure 9b). The cross sections for the triangular and trapezoidal microgrooves in Figure 9b are the same as in Figure 9a. Only the cross section of the rectangular microgrooves is changed with a reduction to a 230 µm width and 200 µm height. This leads to a reduction of liquid film in the microgroove and a reduction of the surface tension effect that increases with the microgroove perimeter. Recall that the perimeter of the triangular and trapezoidal microgrooves is about 3446 µm and that of the rectangular microgroove is about 860 µm. The maximum heat for the triangular microgroove is less than for the trapezoidal one even though their perimeters are the same, because the condensation and evaporation heat transfer are improved in the microgroove shape with more corners. The major part of the liquid film is retained in the corners, reducing the thermal resistance of the liquid film on the groove sides between the corners.

#### 3.1.4. Influence of the Heat Pipe Length 

The influence of the heat pipe adiabatic length is investigated for a fixed length of the evaporator. Once the internal parameters are fixed, it remains to determine the length of the condenser and adiabatic zone. The main difference between these two zones is the presence or absence of fins that enhance the heat transfer between the heat pipe and ambient surroundings. However, the total length is yet to be defined at $L_{tot}=6.3\;\mathrm{cm}$. The total evaporator length is fixed at 1.3 cm. This section deals with the heat pipe performance when the adiabatic length $L_{a}$ varies between 0 and 5 cm. 

**Figure 10:**
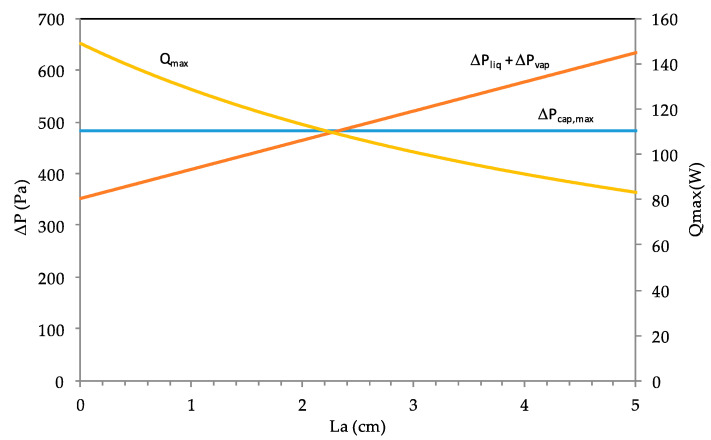
Capillary pressure, differences in liquid pressure, vapor, and maximum heat versus the adiabatic length.

In Figure 10, the liquid and vapor pressure difference as well as the maximum heat are shown versus the adiabatic length for the heat pipe with rectangular microgrooves. Respecting the optimum ranges defined for the rectangular grooves, the depth and width of the grooves of the studied heat pipe are fixed to 800 μm and 270 μm, respectively. The maximum heat is greater when the adiabatic length is short. Indeed, the effective length, defined by $L_{eff}=0.5\left(L_{e}+L_{c}\right)+L_{a}$, increases when the adiabatic length $L_{a}$ increases. The increase of the effective length increases the area of the vapor–liquid heat transfer.

#### 3.1.5. Heat Pipe Heat Transport Limitations

To define the heat pipe operational zone, there are various heat transport limitations defined in the previous work, taking into account the hydrodynamic and thermal processes. Each limit should be calculated versus the operating temperature. The region bounded by all the considered limits is used as the heat pipe operational zone. These limits depend on the two-phase flow, heat flux, heat pipe geometry and size, capillary structure, and the thermophysical properties of the working fluid. Additionally, to the capillary limitation, there are the viscous, boiling, driving, and sonic limitations. These limitations must be taken into account when defining the heat pipe operating temperature and its maximum heat transport.

Viscous limitation: The viscous limitation is reached at a low operating temperature at which the evaporator pressure is very small and could be balanced by the viscous forces that dominate the vapor flow. The viscous limitation occurs for longer heat pipes where the vapor pressure is not sufficient to drive flow from the evaporator to the condenser. Busse [17] showed that for a zero-condenser pressure, the heat pipe viscous limitation is reached, and the following expression is used:

(41) $$Q_{visq}=\frac{r_{hv}r_{hv}h_{fg}\rho_{v}P_{v}\;A_{v}}{16\;\mu_{v}\;l_{eff}}$$

where $P_{v}$ and $\rho_{v}$ are the vapor pressure and density, respectively, and $r_{hv}$ is the heat pipe vapor hydraulic radius.

Sonic limitation: This limit can be reached when the vapor pressure becomes very low and a significant amplification of the vapor velocity is attained at very high heat fluxes. The expression for the sonic limit is defined to be

(42) $$Q_{son}\;=\;A_{v}\rho_{v}h_{fg}\sqrt{\gamma R_{v}T_{v}}$$

where *A_v_* is the cross-sectional area of the vapor flow, $\rho_{v}$ is the vapor density, *P_v_* is the vapor pressure, $h_{lv}$ is the latent heat, and γ is the heat capacity ratio at constant pressure and volume.

Entrainment limit: In the heat pipe, liquid and vapor flow in counter current directions. At high velocity, micro-droplet entrainment occurs resulting from interfacial interactions of the vapor and liquid phases. The maximum entrainment heat capacity can be defined [18] as

(43) $$Q_{ent}=A_{v}h_{fg}\;\sqrt{\frac{\;\rho_{v}\;\sigma }{2\;R_{m}}}$$

where *R_m_* is the average capillary radius.

Boiling limitation: This limit characterizes the evaporator dry-out that occurs when vapor resulting from nucleate boiling blocks the liquid from wetting the heat exchange surface. The heat flux characterizing this limit is defined by

(44) $$Q_{boiling}\;=\frac{k_{eff}\;L_{eff}\;W_{v}}{D_{hv}}\frac{2\;\sigma \;T_{v}}{\rho_{v}\;h_{lv}}\left(\frac{1}{R_{b}}-\frac{1}{R_{m}}\right)$$

where $k_{eff}$ is the effective thermal conductivity; $L_{eff}$ is the effective length; *R_m_* is the capillary radius; and $R_{b}$ is the bubble radius, strongly dependent on the experimental conditions on the heat pipe (presence of non-condensable gas, surface state at the solid–liquid interface, etc.).

**Figure 11:**
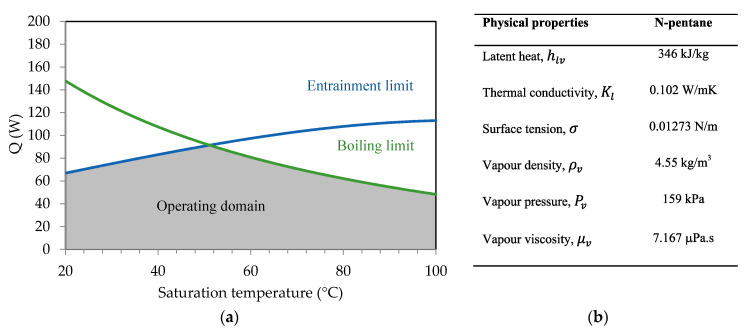
*N*-Pentane: (**a**) heat transport limitations, (**b**) physical properties.

For a flat heat pipe, Figure 11 shows the heat transport limitations versus the saturation temperature. Calculations are conducted for *n*-pentane as a working fluid. Estimations of the viscous, sonic, entrainment, and boiling limitations are conducted using the various parameters of the flat heat pipe defined in Table 1. These parameters are obtained using an evaporator length of 13 mm, condenser length of 30 mm, and adiabatic length of 30 mm. The capillary radius is determined using the maximum capillary pressure defined in Figure 10. $D_{hv}$ and $A_{v}$ are calculated using the equations defined in Section 2.2.2. The working fluid physical properties are estimated as a function of the saturation temperature. Calculations show that the viscous and sonic limitations are the highest values for all the range of the tested vapor saturation temperatures. Entrainment and boiling limits are the lowest power defining the operational zone for the heat pipe. For the entire saturation temperature range, the heat pipe maximum heat is about 100 W when using *n*-pentane as a working fluid.

**Figure 12:**
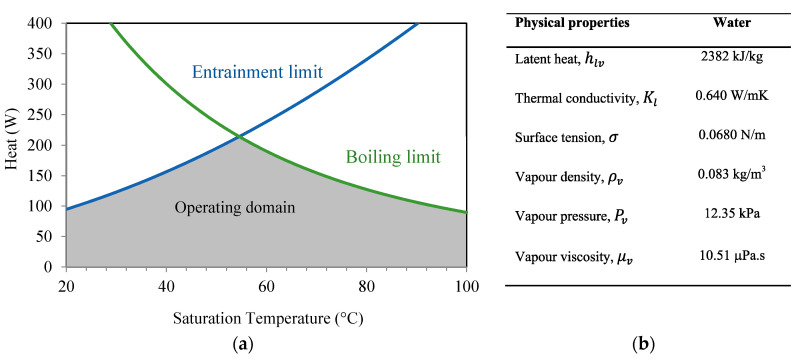
Water: (**a**) heat transport limitation, (**b**) physical properties.

Respecting the capillary limit, Figure 11 and Figure 12 show the operating domains of the studied flat heat pipe as a function of the operating temperatures using water or *n*-pentane as the working fluid. In these two figures, the entrainment and boiling limitations are plotted and delimit the operating domain (the gray area in each figure). The sonic and viscous limitations are very high compared with the boiling and entrainment limitations. Using water as a working fluid, the sonic limitation is 10^3^–2.10^4^ W and the entrainment limitation is 10^3^–5.10^3^ W. For *n*-pentane, the sonic limitation is increased to 6.10^3^–3.10^4^ W and the entrainment limitation is 800–1200 W. In the operating range, the maximum heat of the studied flat heat pipe is defined as a function of the flow temperature. For the operating temperature of 55 °C, the maximum heat is about 80 W when using *n*-pentane as working fluid and 200 W when using water. The maximum heat is approximately two times higher for water than for *n*-pentane due to the water latent heat, which is 7 times higher than that of *n*-pentane.

### 3.2. Experimental Tests on the Optimal Flat Heat Pipe

**Figure 13:**
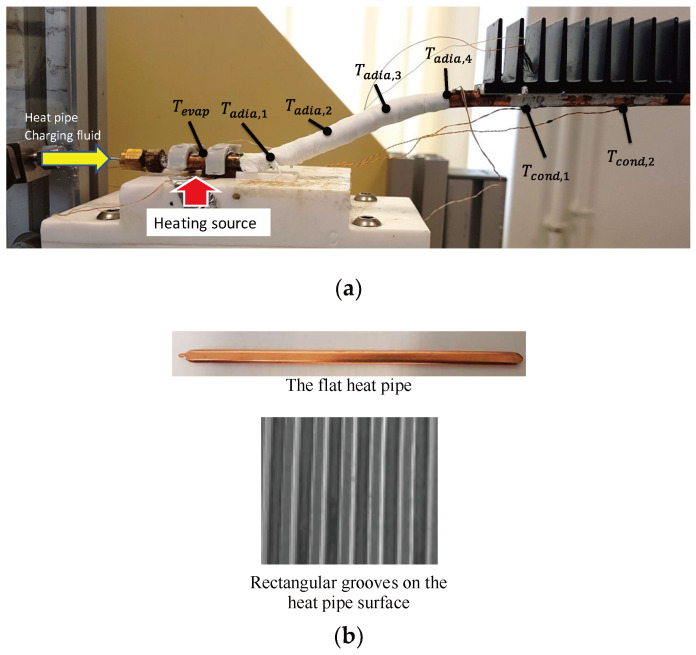
The tested flat heat pipe: (**a**) instrumentation, (**b**) microgrooves.

In this section, the performance of the heat pipe optimum configuration is investigated. Experiments are conducted with a flat heat pipe fabricated using a copper tube with 1 mm thickness, 10 mm width, and 5 mm height. Rectangular microgrooves, 775 μm in depth and 220 μm in width, are used as a capillary wick in the heat pipe. The grooves’ size and shape are defined as the optimum parameters based on the results obtained from the analytical model. Figure 13b shows an image of the rectangular microgrooves inside the heat pipe obtained by Scanning Electron Microscopy (SEM). Figure 13a shows a photo of the studied flat heat pipe with 7 K-type thermocouples soldered on its surface to measure the wall temperatures of the evaporator, adiabatic zone, and condenser. All the thermocouples were calibrated with an accuracy of 0.1 °C. Deionized water was used as the working fluid. The heat pipe was charged with 100% of the microgroove void volume. The vapor cross section is 46% of the total heat pipe cross section. The liquid cross section is 26% of the total heat pipe cross section. A Labview data acquisition system was used to record temperature evolution over time. A numerical wattmeter was used to measure the heat flux imposed by the heat source.

**Figure 14:**
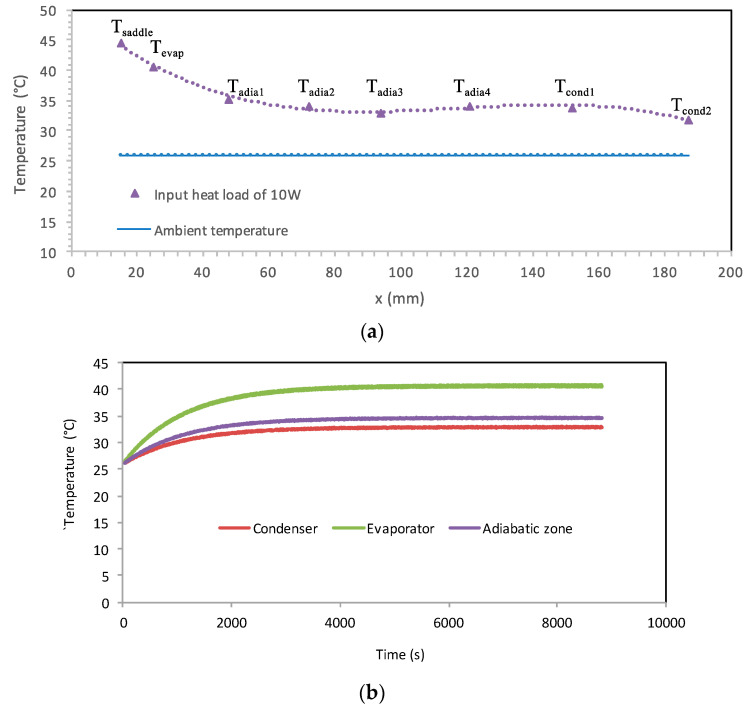
The heat pipe temperatures: (**a**) steady distribution, (**b**) transient responses.

Experiments were conducted for different values of the input heat loads. Figure 14a shows the distribution of the steady state temperatures measured along the heat pipe for a 10 W input heat load. The first temperature is obtained by the thermocouple located inside the cooper saddle. Four thermocouples (*T_adia_*_1_, *T_adia_*_2_, *T_adia_*_3_, *T_adia_*_4_) are used to measure the temperatures in the adiabatic zone between the evaporator and condenser. The lowest temperatures in the heat pipe are measured at the condenser zone by the thermocouples *T_cond_*_1_ and *T_cond_*_2_. Only one thermocouple is located in a position to measure the evaporator temperature (*T_evap_*). Temperatures measured along the adiabatic zone are approximately equivalent because the heat loss to the ambient surroundings is negligible. Figure 14b shows the evaporator, adiabatic zone, and condenser. The transient adiabatic temperature is the average value of *T_adia_*_1_, *T_adia_*_2_, *T_adia_*_3_, and *T_adia_*_4_. The transient condenser temperature is the average response of the thermocouples *T_cond_*_1_ and *T_cond_*_2_. Moreover, all the measured temperatures increase over time due to the continuous heating source. During the transient step, the measured temperatures are variable against time because the mass flow rate coming from the evaporator to the condenser is lower than that leaving the condenser. At *t* = 3000 s, the two-phase flow in the heat pipe tends to be stable and reaches a steady state where the liquid mass flow rate becomes equivalent to the vapor mass flow rate. The wall temperatures and, consequently, the heat pipe pressures become stable over time.

The studied heat pipe is viewed as a series of thermal resistances in order to analyze the steady heat transfer inside it. The first resistance is the heat pipe thermal resistance, defined as the ratio between the difference of the evaporator and condenser temperatures and the input heat load:

(45) $$R_{HP}=\frac{T_{evap}-T_{cond}}{Q_{input}}.$$

The system thermal resistance is generally used as an index for choosing efficient cooling technology. The lower this resistance is, the better it is. For the same input heat, the difference between the evaporation and ambient temperatures decreases when the system thermal resistance is reduced. $R_{syst}$ is expressed as

(46) $$R_{syst}=\frac{T_{evap}-T_{amb}\;}{Q_{input}}.$$

The condenser thermal resistance is defined as

(47) $$R_{cond}=\frac{T_{cond}-T_{amb}\;}{Q_{input}}$$

where $T_{cond}$ is the condenser average temperature, $T_{evap}$ is the evaporator average temperature, and $T_{amb}$ is the ambient temperature.

**Figure 15:**
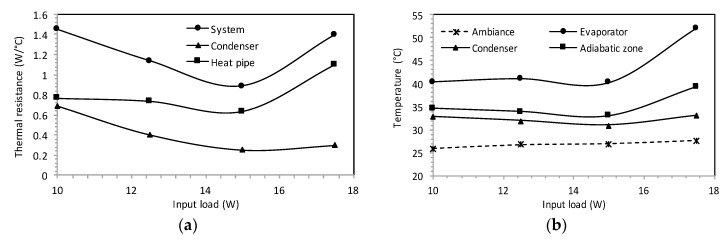
Performance of the flat heat pipe: (**a**) thermal resistances, (**b**) temperatures.

Figure 15a,b show the thermal resistances and the measured steady state temperatures against the input heat load. It is noted that the thermal resistances have the lowest values for the input heat—approximately 15 W—according to the analytical results. The thermal resistances increase for low input heat load ($Q_{input}$ < 14 W) where the liquid fill depth in the microgrooves is deeper than for a heat load between 14 and 16 W; it increases the thermal resistances of heat transfer. For high input heat load ($Q_{input}$ > 16 W), the liquid thickness is very small in the microgrooves and becomes insufficient to dissipate heat by vaporization. In this case, the thermal resistance and temperature increase as shown in Figure 15a,b. Figure 15b shows the heat pipe operating temperature evolution under the variation of the input heat load. The operating temperature is equivalent to that of the adiabatic zone. It can be seen that there is no significant impact on the operating temperature for the low input heat load.

## 4. Conclusions

An analytical model for a flat heat pipe with axial microgrooves was developed to predict the heat transport limitations and maximum heat. Numerous parameters influencing the thermal performance of the flat heat pipe with microgrooves were studied and analyzed. The conclusions can be summarized as follows:

(a) The effect of microgroove width and depth for a flat heat pipe was investigated. It can be seen that increasing microgroove width increases the maximum heat for low values of width and decreases it for high width values. An optimal width and depth were defined for the highest maximum heat.
(b) For the flat heat pipe with trapezoidal microgrooves, the impact of the inclination angle (β) of the groove side slope was analyzed for different heat pipe depths. The maximum heat is influenced by β for low values of the microgroove depth. For high values of *h_g_* (>800 μm), the angle β has no effect on the maximum heat flux.
(c) The influence of microgroove shape on the heat pipe efficiency is highlighted by studying different architecture of grooves respecting the same cross-sectional area or the same hydraulic diameter. For the same microgroove cross sections, the lowest heat transfer was obtained by microgrooves with a triangular cross section. The highest maximum heat was obtained for the rectangular microgroove cross section. For the same hydraulic diameter, the best configuration is the trapezoidal cross section, for which the highest maximum heat was obtained.
(d) Heat transport limitations were studied versus operating temperature, taking into account the hydrodynamic and thermal processes inside the heat pipe. Respecting the capillary limit, entrainment and boiling limits delimited the operating domain. The maximum heat is approximately 2 times higher for water than for *n*-pentane due to the water latent heat, which is 7 times higher than that of *n*-pentane.
(e) Experiments were conducted using a flat heat pipe respecting the optimal geometrical parameters defined by the analytical model. DI water was used as the working fluid. Measurements of thermal resistances and temperatures were in accordance with the performance predicted by the model.

## Figures and Tables

**Table 1:** Heat pipe parameters.

Parameters	Values
Hydraulic vapor diameter, $D_{hv}$	$1.62\;10^{-2}\;m$
Effective length, $L_{eff}$	$4.15\;10^{-2}\;m$
Capillary radius, $R_{m}$	$2.86\;10^{-4}$ m
Nucleation radius, $R_{b}$ [18]	$5\;10^{-4}\;m$
Vapor flow cross section, $A_{v}$	$6\;10^{-5}\;m$^2^
Width of vapor cross section, $W_{v}$	$10^{-2}$ m
